# Metabolic Analysis of DFO-Resistant Huh7 Cells and Identification of Targets for Combination Therapy

**DOI:** 10.3390/metabo13101073

**Published:** 2023-10-12

**Authors:** Koichi Fujisawa, Toshihiko Matsumoto, Naoki Yamamoto, Takahiro Yamasaki, Taro Takami

**Affiliations:** 1 Department of Environmental Oncology, Institute of Industrial Ecological Sciences, University of Occupational and Environmental Health, 1-1 Iseigaoka, Yahatanishi-ku, Kitakyushu 807-8555, Japan; 2 Department of Gastroenterology and Hepatology, Graduate School of Medicine, Yamaguchi University, Minami Kogushi 1-1-1, Ube, Yamaguchi 755-8505, Japan; tm0831@yamaguchi-u.ac.jp (T.M.); nao-yama@yamaguchi-u.ac.jp (N.Y.); t-takami@yamaguchi-u.ac.jp (T.T.); 3 Amaguchi University Health Administration Center, 1677-1 Yoshida, Yamaguchi 753-8511, Japan; 4 Department of Oncology and Laboratory Medicine, Graduate School of Medicine, Yamaguchi University, Minami Kogushi 1-1-1, Ube, Yamaguchi 755-8505, Japan; t.yama@yamaguchi-u.ac.jp

**Keywords:** hypoxia, iron chelator, energy metabolisms, autophagy, liver cancer, glutamine, metabolomic analysis, combination therapy, autophagy inhibitor, glutaminase inhibitor

## Abstract

Hepatocellular carcinoma (HCC) is one of the most refractory cancers with a high rate of recurrence. Iron is an essential trace element, and iron chelation has garnered attention as a novel therapeutic strategy for cancer. Since intracellular metabolism is significantly altered by inhibiting various proteins by iron chelation, we investigated combination anticancer therapy targeting metabolic changes that are forcibly modified by iron chelator administration. The deferoxamine (DFO)-resistant cell lines were established by gradually increasing the DFO concentration. Metabolomic analysis was conducted to evaluate the metabolic alterations induced by DFO administration, aiming to elucidate the resistance mechanism in DFO-resistant strains and identify potential novel therapeutic targets. Metabolom analysis of the DFO-resistant Huh7 cells revealed enhanced glycolysis and salvage cycle, alternations in glutamine metabolism, and accumulation of dipeptides. Huh7 cultured in the absence of glutamine showed enhanced sensitivity to DFO, and glutaminase inhibitor (CB839) showed a synergistic effect with DFO. Furthermore, the effect of DFO was enhanced by an autophagy inhibitor (chloroquine) in vitro. DFO-induced metabolic changes are specific targets for the development of efficient anticancer combinatorial therapies using DFO. These findings will be useful for the development of new cancer therapeutics in refractory liver cancer.

## 1. Introduction

In recent years, cancer has become a significant disease that is the leading cause of death in many countries. Hepatocellular carcinoma (HCC) is one of the most common cancers in the world and one of the leading causes of cancer-related deaths worldwide. Recent advances in therapeutic techniques have improved the prognosis of this malignancy, but the prognosis for patients with advanced hepatocellular carcinoma, especially in the presence of vascular invasion and extrahepatic metastasis, remains poor [1,2]. 

Iron is an important element in the body and many iron-bearing proteins exist [3] and maintaining appropriate iron concentrations is of utmost importance. Specifically, within the 2-oxoglutarate dioxygenase family, various enzymes stand out, including HIF-hydroxylase, which participates in hypoxia response and collagen hydroxylase, and is involved in collagen maturation and the enzymes responsible for carnitine synthesis. Furthermore, other significant iron-sulfur enzymes include ribonucleotide reductase in nucleotide synthesis, mitochondrial aconitase in the TCA cycle, NADH-coenzyme Q reductase in the electron transfer system, and the Rieske protein. Iron metabolism in cancer cells has received much attention in recent years, and its relationship with oxidative stress has been reported [4,5]. Since cancer cells grow faster and require more iron than normal cells, iron control could be a novel cancer therapy [6,7]. One of the most widely recognized iron chelators is deferoxamine (DFO), derived from Streptomyces pilus [8]. The administration of DFO inhibits the activity of iron-requiring enzymes in cells. Iron chelators chelate iron, a cofactor of ribonucleotide reductase (RR), which is involved in DNA synthesis, and reduces RR activity, thereby regulating DNA synthesis in cancer cells and exerting an anti-tumor effect [9]. We have previously shown the suppression of preneoplastic lesion growth in rat livers by DFO [10] and the clinical efficacy of iron chelator DFO in refractory advanced liver cancer. We have demonstrated the effectiveness of the iron chelator deferoxamine, an alternative therapy, for advanced HCC patients with compromised liver function, indicating potential future therapeutic applications for iron chelators [11]. Due to the need for hospitalization for intravenous administration of DFO, we evaluated deferasirox (DFX), a next-generation iron chelator that can be administered orally and treated on an outpatient basis, and we reported that DFX has a concentration-dependent antitumor effect on pancreatic cancer cell lines. We have previously assessed the impact of DFX in HCC and, while its effectiveness was evident in basic research, clinical study did not yield substantial outcomes due to its dose-limiting toxicities. Therefore, new approaches aimed at achieving greater effectiveness by utilizing smaller amounts of iron chelator are being anticipated. 

Combination therapy with iron chelators and arsenic trioxide has been reported to have a synergistic effect on leukemia cells without side effects [12]. In addition, several cell lines have shown that doxorubicin, cisplatin, and carboplatin, in combination with DFX, inhibit cancer cell growth and induce apoptosis [13,14]. Since RR is also involved in gemcitabine (GEM) sensitivity, we reported that combining DFX with GEM not only added the antitumor effect of DFX itself, but also improved GEM sensitivity in pancreatic cancer cell lines [15]. However, there are still combination therapies with DFO that are not yet known, and the search for new drugs is expected.

Since there are many targets, such as various iron-containing proteins, it seems difficult to identify the effects of DFO in detail, and it is necessary to identify which targets are important for each cell type. We have recently reported that DFO triggered a metabolic shift toward glycolysis and increased lactate production in cells, and a synergistic effect of DFO and a lactate excretion inhibitor was observed in HeLa cells; however, the same was not observed in the human liver cancer cell line Huh7 [16]. In this study, we generated a DFO-resistant strain of Huh7 cells and conducted an analysis of the metabolic changes in the presence and absence of DFO. The findings of this study on the combination therapy by forcibly modifying the metabolism through DFO administration will be beneficial for new cancer chemotherapy in the future.

## 2. Materials and Methods

### 2.1. Cell Culture

Huh7 cells (JCRB0403) were procured from the Japanese Collection of Research Bioresources (Osaka, Japan) and cultured in Dulbecco’s Modified Eagle Medium (DMEM) (Life Technologies, Tokyo, Japan), supplemented with 10% fetal bovine serum (SAFC, St. Louis, MO, USA), penicillin (100 U/mL; Life Technologies) and streptomycin (100 μg/mL; Life Technologies) at 37 °C and 5% CO2. Cell passages in cell culture were conducted when the cells reached approximately 80% confluence. Adherent cells were dissociated with 0.05% trypsin-ethylenediaminetetraacetic acid (Life Technologies, Tokyo, Japan) and resuspended in Dulbecco’s modified Eagle’s medium (DMEM; Life Technologies, Tokyo, Japan) containing 10% FBS. Cell confluence was evaluated using an imaging system coupled to a microscope (Biorevo BZ9000; Keyence, Osaka, Japan). After seeding, non-adherent cells were removed when the medium was replaced. The culture medium was changed every 2 days. The establishment of DFO-resistant cell lines involved a gradual increase in DFO concentration (initially starting from 0 µM) over approximately 6 months. The parental cells and obtained cells (approximately 6000 cells per well) were seeded in 96-well plates (351172; Corning, Tokyo, Japan) and subjected to various concentrations (0 to 100 µM) of DFO. Cell proliferation was quantified by assessing cell area through the Incucyte HD imaging system (Essen BioScience, Ann Arbor, MI, USA) (*n* = 6, three independent experiments were performed). The parental cells were seeded in 96-well plates (approximately 2000 cells per well) and subjected to various concentrations of drugs. The synergy between the drugs was evaluated using the CalcuSyn software program (Biosoft, Ferguson, MO, USA), which calculates the combination index (CI) to quantify the level of drug interactions. The DFO was purchased from Novartis Pharma K.K. (Tokyo, Japan, D01186). The CB839 and CQ were purchased from Cayman chemical Co. (Ann Arbor, MI, USA, No22038) and Fujifilm (Tokyo, Japan, 036-17972), respectively.

### 2.2. Metabolome Analysis

Cells used for the metabolomic analysis were cultured in a 10 cm dish until Huh7 reached 70% confluence, and the DFO was added to the medium to reach the desired concentration. Three days after DFO addition, adherent cells were detached and collected, washed 3 times with PBS, stored at −80 °C, and sent to Metabolon Inc. for further analysis (*n* = 3). Metabolomic and statistical analyses were carried out at Metabolon (Morrisville, NC, USA), following previously described methods [17,18]. In summary, cellular pellets (5 × 10^5^ cells) were subjected to methanol extraction. The resulting extract was then subdivided into portions for analysis using ultrahigh performance liquid chromatography/mass spectrometry (UHPLC/MS,) (Thermo Scientific, Waltham, MA, USA) in positive, negative, or polar ion modes, as well as gas chromatography/mass spectrometry (GC/MS) (Thermo Scientific, Waltham, MA, USA). Identification of metabolites was accomplished by matching ion features against a reference library of chemical standards using an automated process, which was subsequently followed by visual inspection to ensure quality control. For statistical computations and data presentation, any absent values were treated as being below the detection thresholds, and these values were estimated using the lowest compound value available. 

### 2.3. Pathway Analysis with Ingenuity Pathways Analysis (IPA)

Pathway analysis was carried out using Ingenuity Pathway Analysis (IPA) (Qiagen, Inc., Germantown, MD, USA). Canonical pathways can additionally be ranked based on the ratio value, which is calculated by dividing the count of molecules in a specific pathway that satisfy defined criteria by the total count of molecules comprising that pathway. Similarly, an upstream regulator analysis was performed to explain observed changes in gene expression within the dataset and unveil the biological activities occurring in the cells under study. This analysis of upstream regulators was carried out using the features of IPA. The upstream regulator analysis serves as a tool for predicting transcriptional regulators and their potential roles, including identifying which regulators might be involved and whether they could be activated. By conducting this analysis using IPA, insights were gained into how regulators and targets interact in this study, providing testable hypotheses for gene regulatory networks.

### 2.4. Statistical Analysis

The analysis of the results was carried out using either the two-tailed unpaired Student’s *t*-test or Welsh’s two-factor *t*-tests. The presented data reflect the mean value along with the standard deviation, and the level of significance was set at *p* < 0.05. In cases where more than two groups were compared, a one-way ANOVA followed by Tukey’s post-hoc test was employed. For evaluating the significance of the metabolomic analysis, a comparison of protein-normalized data between experimental groups was conducted using either ArrayStudio (Omicsoft, Cary, NC, USA or the “R” programming language, with significance denoted by *p* < 0.05. ANOVA contrasts were utilized to detect significant variations in biochemicals among the experimental groups.

## 3. Results

### 3.1. Enhanced Glycolysis and Salvage Cycle, Altered Glutamine Metabolism, and Accumulation of Dipeptides Occur in DFO-Resistant Huh7 Cells

**Figure 1:**
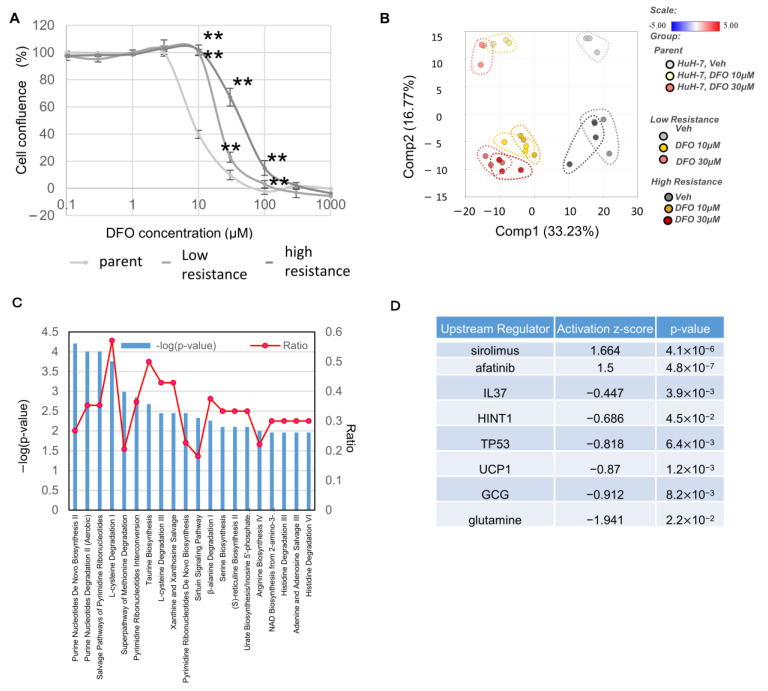
Analysis of metabolites in DFO-resistant Huh7 cell lines. (**A**) Evaluation of DFO-resistant cell lines—evaluation of the degree of resistance in two cell lines, namely a low resistance cell line and high resistance cell line (** *p* < 0.01, One way ANOVA followed by Tukey’s post-hoc test, *n* = 6). Three independent experiments were performed. The establishment of DFO-resistant cell lines involved a gradual increase in DFO concentration (initially starting from 0 µM) over approximately 6 months. (**B**) The cells used for metabolomic analysis were cultured in a 10 cm dish until Huh7 reached 70% confluence, and DFO was added to the medium to reach the desired concentration. Three days after DFO addition, adherent cells were detached and collected, washed 3 times with PBS, stored at −80 °C, and sent to Metabolon Inc. for further analysis. The principal component analysis normalized metabolic data. Percentage values indicated on the axes represent the contribution rate of the first (PC1) and second (PC2) principal components to the total amount of variation. (**C**) Pathway ranking by IPA analysis. Reciprocal display of *p*-value was calculated by IPA software, the orange line is the ratio of metabolites included in each pathway. (**D**) Showing upstream regulators from IPA analysis where activation Z-scores are available. Ingenuity’s Upstream Regulator Analysis was performed in IPA. (This is a tool that predicts upstream regulators from gene expression data based on the literature and compiled in the Ingenuity Knowledge Base.).

We generated a DFO-resistant Huh7 strain to examine the metabolic changes in the Huh7 cell line; two resistance lines were generated, a low-resistance cell line (exhibiting mild DFO resistance) and a high-resistance cell line (exhibiting high DFO resistance). Although the IC50 of DFO for the parent cell line was 7.6 μM, it had an IC50 of 19.1 μM for the low-resistance cell line, and an IC50 of 45.8 μM for the high-resistance cell line (Figure 1A). Next, we decided to evaluate the metabolic changes in response to DFO in parent cells and DFO-resistant cells by metabolomic analysis. Totals of 0 µM, 10 µM and 30 µM were selected from the concentrations used in Figure 1A. The cell lines were clearly distinguished upon the PCA analysis of metabolomic data (Figure 1B). Canonical pathway analysis was performed to determine the most significantly affected pathways and those that are predicted to be activated or inhibited. An IPA of parent cell line and high-resistance cell line revealed an increase in terms associated with canonical pathways related to de novo purine and pyrimidine nucleotide biosynthesis, pyrimidine ribonucleotide salvage pathways, and pyrimidine ribonucleotide interconversion (Figure 1C). An upstream analysis was conducted to investigate the potential control of biological processes, pathways, and diseases by transcriptional regulators and their targets, as well as to examine how these upstream molecules regulate each other’s activities. In addition to sirolimus, which inhibits the mTOR (mechanistic Target of Rapamycin) pathway, EGFR inhibitors afatinib and glutamine were found in upstream regulators analysis (Figure 1D). We included a heatmap of statistically significant biochemicals in Supplementary Table S1.

**Figure 2:**
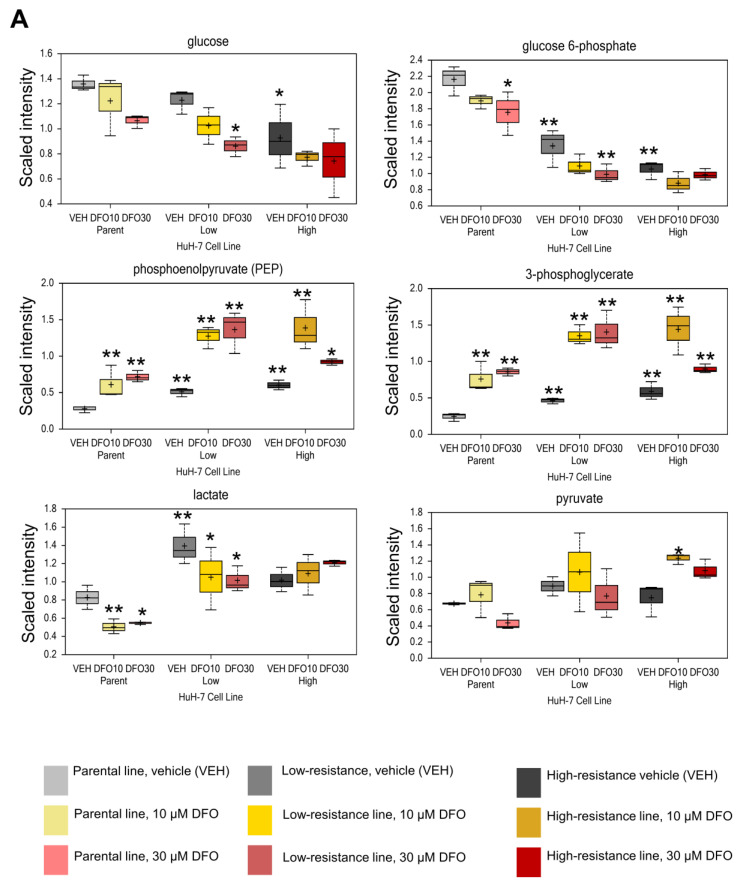

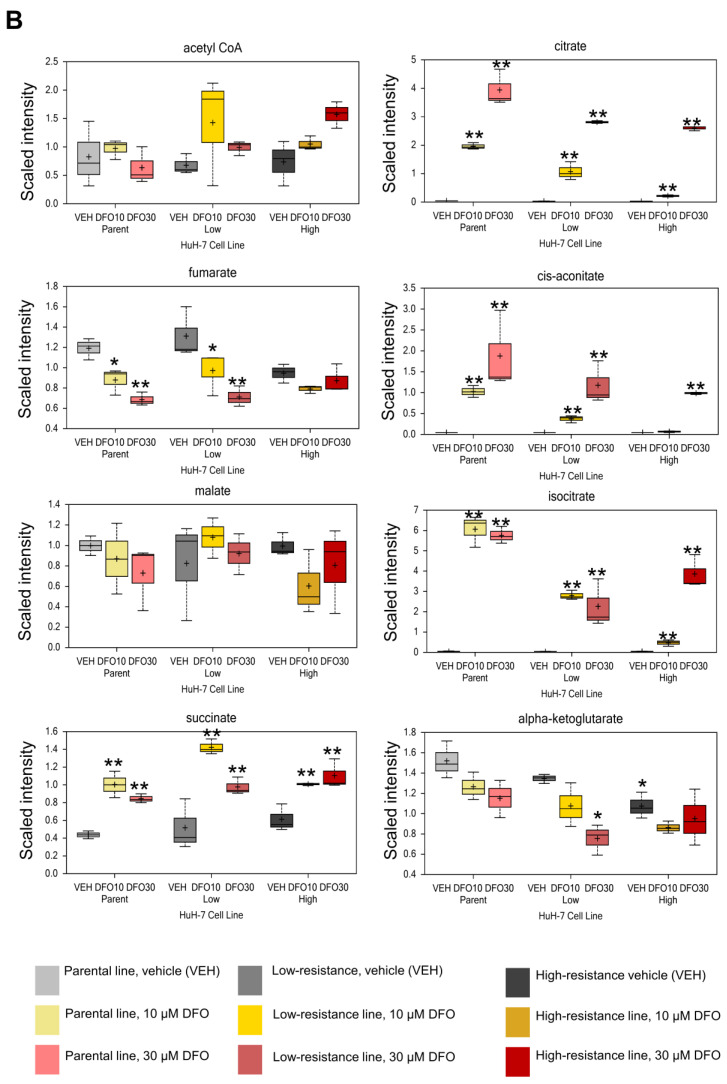

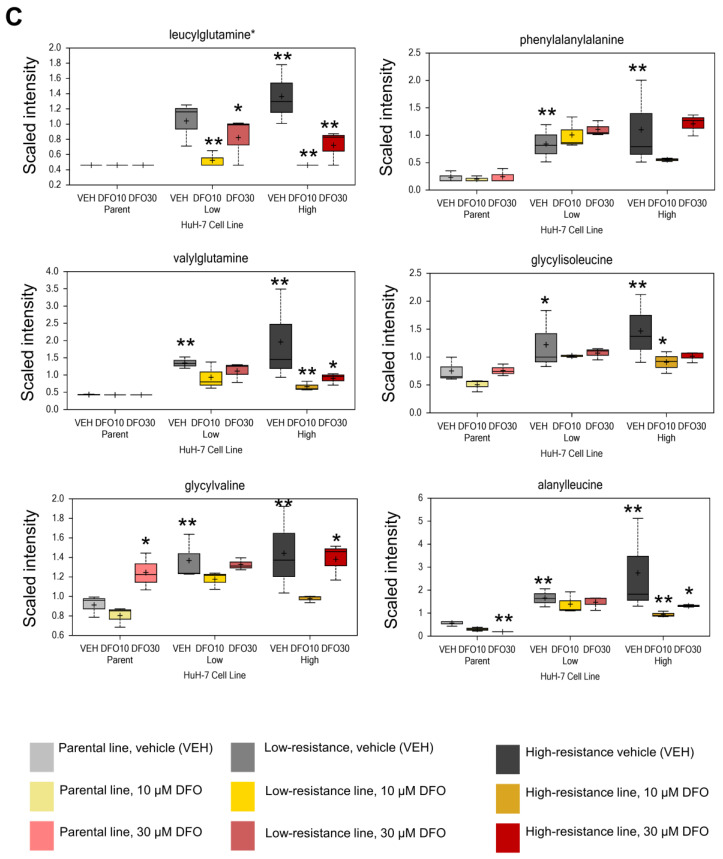
Metabolite changes of glycolysis, TCA cycle, and dipeptides in parent and resistant Huh7. (**A**) Changes in glycolytic metabolites on Day 3 after DFO administration. (**B**) Changes in TCA cycle metabolites on Day 3 after DFO administration. (**C**) Changes in various dipeptides levels after DFO administration. The statistical differences (Parent VEH vs. Parent DFO10, Parent VEH vs. Parent DFO30, Parent VEH vs. low resistant VEH, low resistant VEH vs. low resistant DFO10, low resistant VEH vs. low resistant DFO30, Parent VEH vs. high resistant VEH, high resistant VEH vs. high resistant DFO10, and high resistant VEH vs. high resistant DFO30) were shown by * *p* < 0.05, ** *p* < 0.01 (ANOVA contrasts, *n* = 3).

Furthermore, a comparison of the metabolites revealed elevated phosphoenolpyruvate (PEP) and 3-phosphoglycerate levels in the resistant cell lines in the absence of DFO, which suggested enhanced glycolysis (Parent VEH vs. low VEH and High VEH, Figure 2A). The changes caused by DFO administration included an increase in phosphoenolpyruvate and 3-phosphoglycerate levels, observed in all groups: Parent, Res10, and Res30. However, lactate levels did not increase; instead, they decreased in the parent and low groups (Parent VEH vs. Parent DFO10, Parent VEH vs. Parent DFO30, Low VEH vs. low DFO10, low VEH vs. Low DFO30, Figure 2A). Increased citrate, cis-aconitate, and isocitrate levels—TCA cycle metabolites—were observed in the resistant cell lines after DFO treatment (VEH vs. DFO10, VEH vs. DFO30, Figure 2B). Notably, large amounts of dipeptides, such as leucylglutamine, valylglutamine, and valyleucine also accumulated in the resistant cell lines (Parent VEH vs. Low, High VEH, Figure 2C). 

### 3.2. DFO and GLS Inhibitor Exhibit Synergistic Effects in Huh7 Cells

**Figure 3:**
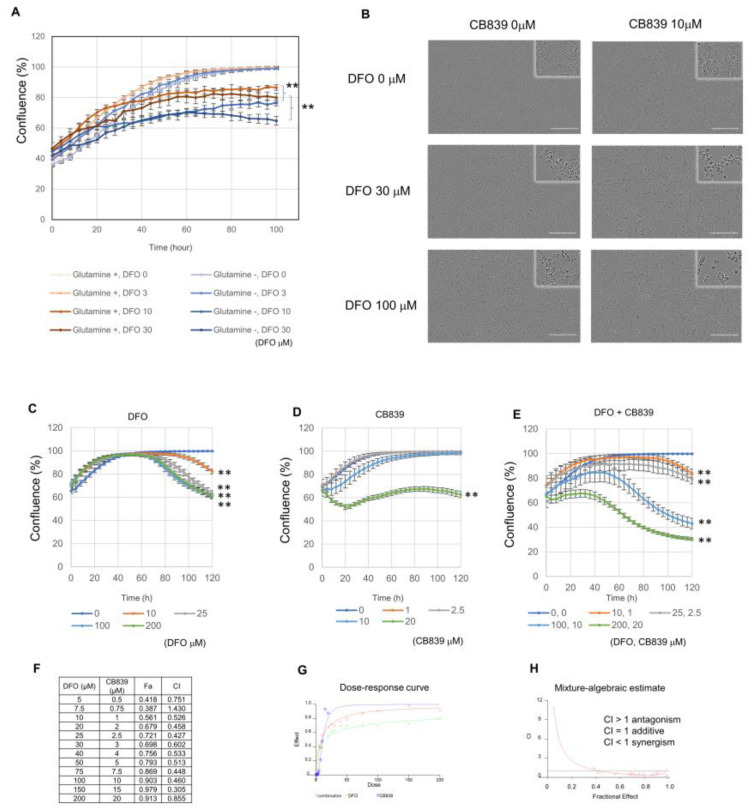
Evaluation of the effect of glutaminase inhibitor (CB839) on Huh7. (**A**) Evaluation of the effect of DFO on Huh7 proliferation in the presence and absence of glutamine administration. The statistical significance was indicated by ** *p* < 0.01 (Student’s *t*-test, *n* = 6). Three independent experiments were performed. (**B**) Alterations observed in cell culture due to the DFO + CB839 treatment, 72 h after administration. The scale bar represents 300 µm. Enlarged picture is shown on the upper right. (**C**) Changes in cell confluence under DFO monotherapy. The statistical significance was indicated by ** *p* < 0.01 compared to DFO 0 μM (One way ANOVA followed by Tukey’s post-hoc test, *n* = 6). Three independent experiments were performed. (**D**) Changes in cell confluence under CB839 monotherapy. The statistical significance was indicated by ** *p* < 0.01 compared to DFO 0μM (One way ANOVA followed by Tukey’s post-hoc test, *n* = 6). Three independent experiments were performed. (**E**) Changes in cell confluence under the DFO + CB839 treatment. The statistical significance was indicated by ** *p* < 0.01 compared to DFO 0 μM (One way ANOVA followed by Tukey’s post-hoc test, *n* = 6). Three independent experiments were performed. (**F**) Combination index of DFO + CB839, where Fa represents the fraction affected and CI indicates the combination index. (**G**) Dose–response curve of the DFO + CB839 treatment. (**H**) Mixture-Algebraic assessment of the DFO + CB839 treatment. Drug interactions were determined using the isobologram and combination index method derived from the median-effect principle of Chou and Talalay (Calcu Syn software, Biosoft, Cambridge, UK).

As glutamine (Gln) was found in upstream regulator analysis, we evaluated the impact of Gln on DFO sensitivity. DFO sensitivity was increased in the non-Gln group compared to that in the Gln group (Figure 3A). This fact suggests that glutaminolysis is related to DFO sensitivity. The subsequent combinatorial treatment with DFO with CB839—a small molecule inhibitor of glutaminase—resulted in a synergistic effect (Figure 3B). In order to investigate this synergistic effect in detail, we evaluated cell proliferation at various concentrations of DFO, CB839, and DFO + CB839 (Figure 3C–E). The combination index of DFO with CB839, the dose-response curve of the DFO with CB839 treatment, and the mixture-algebraic evaluation of the DFO + CB839 treatment were assessed using the CalcuSyn software (Figure 3F–H). The combination indices (CI) were below 1.0, suggesting that DFO and CB839 act in a synergistic manner.

### 3.3. DFO and Autophagy Inhibitors Exhibit a Synergistic Effect in Huh7 Cells

**Figure 4:**
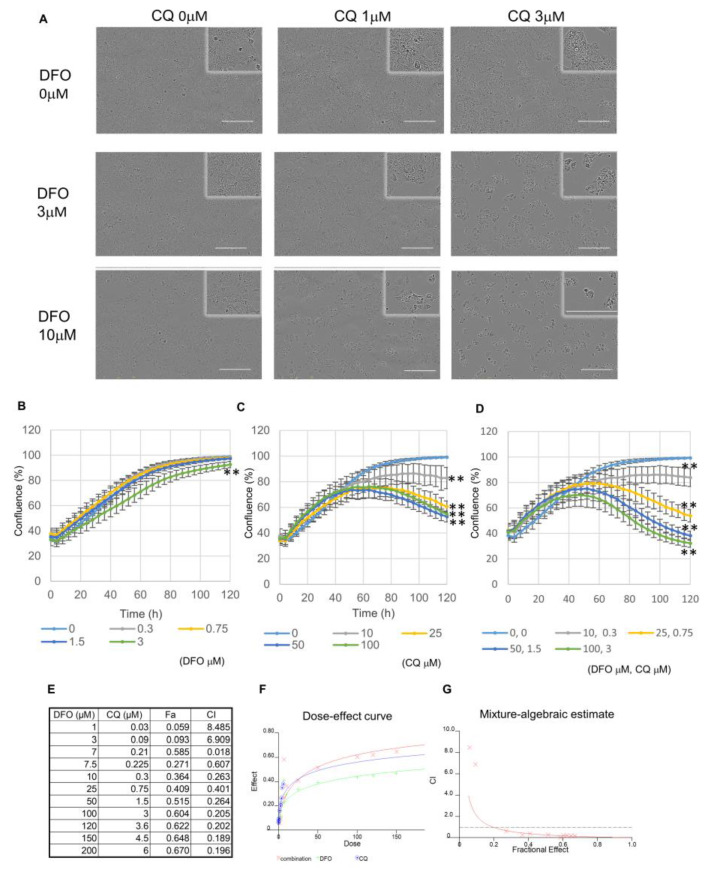
Evaluation of the effect of DFO + CQ (autophagy inhibitor). (**A**) Evaluation of the effect of DFO on Huh7 in the presence and absence of CQ administration. Changes seen in cells from the DFO + CQ treatment—72 h after administration, Scale bar, 300 µm. The enlarged picture is shown on the upper right. (**B**) Changes in cell confluence observed under DFO monotherapy. The statistical significance was indicated by ** *p* < 0.01 compared to DFO 0 μM (One way ANOVA followed by Tukey’s post-hoc test, *n* = 6). Three independent experiments were performed. (**C**) Changes in cell confluence observed under CQ monotherapy. The statistical significance was indicated by ** *p* < 0.01 compared to DFO 0 μM (One way ANOVA followed by Tukey’s post-hoc test, *n* = 6). Three independent experiments were performed. (**D**) Changes in cell confluence observed under the DFO + CQ treatment. The statistical significance was indicated by ** *p* < 0.01 compared to DFO 0 μM (One way ANOVA followed by Tukey’s post-hoc test, *n* = 6). Three independent experiments were performed. (**E**) Combination index of DFO + CQ, where Fa represents the fraction affected and CI signifies the combination index. (**F**) Dose–response curve of the DFO + CQ treatment. Drug interactions were determined using the isobologram and combination index method derived from the median-effect principle of Chou and Talalay (Calcu Syn software, Biosoft). (**G**) Mixture-Algebraic evaluation of the DFO + CQ treatment.

**Figure 5:**
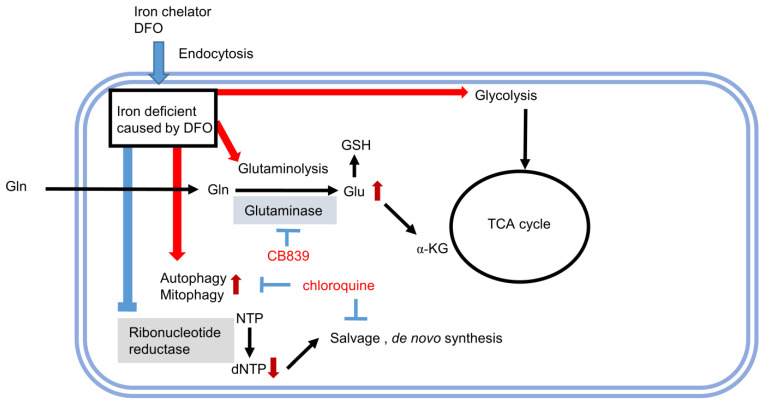
Schematic representation of metabolism altered by DFO administration and combination therapy with specific inhibitors. DFO, an iron chelator, can be taken up into cells by endocytosis. DFO chelates unbound iron in the cytoplasm, thereby reducing the activity of iron-requiring enzymes, such as ribonucleotide reductase (RR) and Prolyl Hydroxylase Domain-Containing Proteins (PHDs). PHDs hydroxylate HIF1α, and when their activity is reduced, HIF1α accumulation occurs, resulting in accumulation of HIF1α and expression of genes involved in hypoxia. Glutamate (Glu) is produced from Glutamine (Gln) by glutaminase and is used for GSH or αKG. CB839 suppresses Gln production, resulting in an antitumor effect. Furthermore, RR with low activity reduces the production of dNTP from NTP and increases the salvaging of purines and pyrimidines to compensate for this decrease. The suppression of autophagy by chloroquine is also effective.

Even though DFO and CB839 act in a synergistic manner, we searched for a drug that can provide an even greater synergistic effect when used in combination with DFO. DFO is suggested to enhance the purine and pyrimidine salvage cycles from the pathway data in our previous report. As these salvage cycles are thought to be involved in autophagy, we evaluated whether DFO induces autophagy in Huh7 cells. Therefore, we examined the effect of combinatorial treatment with DFO and an autophagy inhibitor chloroquine (CQ). In the combinatorial treatment with CQ and DFO, 1 μM CQ resulted in a mild DFO effect and 3 μM produced an even greater DFO effect (Figure 4A). In order to investigate the synergistic effect of DFO and CQ in detail, we evaluated cell proliferation at various concentrations of DFO, CQ, and DFO + CQ (Figure 4B–D). The combination index of DFO with CQ, the dose–-response curve of the DFO with CQ treatment, and the mixture-algebraic evaluation of the DFO + CQ treatment were assessed using the CalcuSyn software (Figure 4E–G). The combination indices (CI) were below 1.0, indicating that DFO and CQ act in a synergistic manner. Figure 5 provides an overview of the metabolic shifts triggered by DFO, along with the corresponding target molecules.

## 4. Discussion

It is important to identify the metabolic changes that can serve as new therapeutic targets among the various metabolic changes that occur in response to the DFO treatment. In this study, we created a previously undocumented cell line resistant to DFO and examined alterations in metabolic processes. Moreover, we evaluated the metabolic changes occurring in these cells in response to the DFO treatment and investigated the metabolic changes that arise in the same cells as they develop resistance against DFO.

Notably, significant accumulation of dipeptides in the DFO-resistant Huh7 cell lines was observed. Previous metabolomic studies on nutrients related to the survival and maintenance of chronic myelogenous leukemia (CML) and hematopoietic stem cells indicated that there is specific accumulation of dipeptides in CML stem cells, and dipeptides are involved in the maintenance of stem cells [19]. Based on these observations, DFO-resistant cell lines are thought to possess more stem cell properties; however, further studies are needed for confirmation. We believe that the upregulation of the salvage cycle and enhanced de novo synthesis of nucleic acids in the DFO-resistant cell line can be explained by a reduced dNTP synthesis by the iron-dependent ribonucleotide reductase (RR) enzyme due to the chelation of iron. In DFO-treated cells, citrate, isocitrate, and cis-aconitate, which are the first three metabolites in the TCA cycle, were increased. Accumulation of HIF1α suppresses mitochondrial metabolism, reduces citrate synthase, aconitase, isocitrate dehydrogenase and succinate dehydrogenase activity after administration of an iron chelator [20]. A stem-loop structure called IRE (iron responsive element) is conserved in the mRNA of aconitase, and an RNA-binding protein called IRP (iron regulatory protein) selectively binds to IRE only under iron deficiency [21]. This is thought to cause stagnation of the TCA cycle. Under hypoxic conditions, a metabolic change known as the reverse TCA cycle occurs, in which alternative fuels such as glutamine are used to induce reductive carboxylation by isocitrate dehydrogenase and other enzymes to isocitrate, cis-aconitate, and citrate [22]. The increase in citrate, isocitrate, and cis-aconitate observed in DFO-treated cells could also be attributed to the activation of the reverse TCA cycle. Similar to DFO-resistant HeLa cell lines [16], Huh7 cells exhibited upregulated salvage cycle and enhanced de novo pathway for the synthesis of purine and pyrimidine nucleotides, pyrimidine ribonucleotides, and pyrimidine nucleotide interconversion. In general, pyrimidine nucleotides are synthesized via the de novo pathway using simple precursors or via the salvage pathway using preformed pyrimidines derived from compounds of endogenous or foreign origin. In addition, Huh7 cells showed enhanced glycolysis, although not as remarkable as the change seen in HeLa cells [16]. In HeLa cells, administration of DFO resulted in an increased emphasis on the glycolytic pathway, leading to elevated lactate levels. This effect was augmented by the co-administration of CHC, which inhibits the pump responsible for extruding lactate from the cells. However, in Huh7 cells, no synergistic effect of DFO and CHC was observed. We concluded that the DFO treatment did not lead to an increase in the expression of LDH, the enzyme responsible for converting pyruvate to lactate in the previous report [19]. Nonetheless, it aligns with the previous report that in both parent and resistant cells, administration of DFO did not lead to an increase in lactate levels. In this study, the lack of lactate increase upon DFO administration in both Huh7 parent cells and resistant cells aligns with the previous report indicating no elevation in LDH expression. Instead of glycolysis, glutamine was identified as an upstream regulator in IPA. In general, cancer cells exhibit enhanced glutaminolysis and glutamine uptake via membrane transporters like SLC1A5 and SLC7A5. Glutaminase catalyzes the production of glutamate from glutamine and supplies the former into the TCA cycle to support the proliferation of cancer cells, and glutamate is further metabolized to the antioxidant peptide glutathione. Cancer cells with an activated NRF2 antioxidant pathway exhibit metabolic imbalances in central carbon metabolism for maintaining increased antioxidant capacity, and this change has been shown to be a potential therapeutic target [23]. CB839, a small molecule inhibitor of glutaminase, is garnering attention due to its potential efficacy in glutamate ‘addicts’ like triple negative breast cancer and renal cell carcinoma [24]. The results of the present study also indicate the importance of glutaminolysis in Huh7 cells, where the effect of DFO was enhanced in cultures without glutamine, and the combinatorial treatment with DFO and the GLS inhibitor CB839 was effective. CB839 is already in the clinical trial stage and may offer new avenues in the context of combinatorial therapy.

In cancer therapy, drug resistance to analogue-based drugs, e.g., gemcitabine and Ara-C, has been reported to be closely associated with purine and pyrimidine salvaging [25]. Drugs based on nucleoside variants have been suggested as potential chemotherapeutic agents for certain types of cancers [26], and its potential value in combination with DFO may be assessed in the future. Ribonucleotide reductase is one of the targets of DFO, and upregulation of the nucleic acid salvage cycle and enhanced de novo synthesis to counteract the reduction in dNTPs after DFO treatment are important processes in ensuring cell survival in response to DFO treatment. It has been reported that autophagy supplies metabolic substrates to maintain the nucleotide pools [27]. As autophagy and dNTP pool levels are regulated by negative feedback, knocking down RR induces autophagy [28]. Known autophagy inhibitors include 3-methyladenine, bafilomycin A, CQ and hydroxychloroquine, etc. [29]. CQ has long been known as an antiparasitic drug, and various reports indicate its anticancer effect based on the inhibition of a wide range of cell remodeling and autophagy functions as a mechanism for quality control [30,31]. This is thought to be very effective in cancer cells that are highly dependent on autophagy. CQ monotherapy in liver cancer cells has been shown to suppress cancer growth both in vitro and in vivo [32]. In liver cancer stem cells, chloroquine has been demonstrated to have therapeutic potential by inhibiting autophagy, which is essential for the survival of cancer stem cells under conditions of oxygen and nutrient deprivation, a prevalent characteristic of the tumor microenvironment [33]. A recent intriguing report was published about sorafenib, the first FDA-approved drug for advanced hepatocellular carcinoma (HCC) treatment. This study suggests that alterations in glucose metabolism prompted by sorafenib play a crucial role as a resistance mechanism in HCC cells. As a metabolic recycling process, autophagy governs various vital pathways related to cell survival and death. Notably, chloroquine-mediated autophagy inhibition has been documented to notably enhance the sensitivity of HCC cells to sorafenib, potentially reversing the enhanced glycolysis [34]. In future study, employing cancer stem cells could potentially be valuable in a combination therapy with CQ, which inhibits autophagy that is upregulated for liver survival in response to the metabolic alterations triggered by DFO administration. Further research is expected to demonstrate that a greater antitumor effect can be achieved by combining DFO and CQ.

## 5. Conclusions

The combination of DFO with either the glutamine synthesis inhibitor (CB839) or the autophagy inhibitor (CQ), both of which are related to the metabolic changes induced by DFO treatment, yielded a synergistic therapeutic effect. Going forward, we expect that combinatorial pharmacotherapeutic cancer treatments that target parts of cell metabolism and are forcibly altered by DFO administration will become a useful strategy.

## Data Availability

The data presented in this study are available as part of Supplementary Data.

## Supplementary Materials

The following supporting information can be downloaded at: https://www.mdpi.com/article/10.3390/metabo13101073/s1, Table S1: Heat map of statistically significant biochemicals, Supplemental data1: all metabolomics analysis data.
